# The Role of Neutrophil Extracellular Traps in Cancer

**DOI:** 10.3389/fonc.2021.714357

**Published:** 2021-08-12

**Authors:** Bo-Zong Shao, Yi Yao, Jin-Ping Li, Ning-Li Chai, En-Qiang Linghu

**Affiliations:** Department of Gastroenterology, General Hospital of the Chinese People’s Liberation Army, Beijing, China

**Keywords:** neutrophil extracellular trap, cancer, neutrophil, immunity, inflammation

## Abstract

Neutrophils are vital components of innate and adaptive immunity. It is widely acknowledged that in various pathological conditions, neutrophils are activated and release condensed DNA strands, triggering the formation of neutrophil extracellular traps (NETs). NETs have been shown to be effective in fighting against microbial infections and modulating the pathogenesis and progression of diseases, including malignant tumors. This review describes the current knowledge on the biological characteristics of NETs. Additionally, the mechanisms of NETs in cancer are discussed, including the involvement of signaling pathways and the crosstalk between other cancer-related mechanisms, including inflammasomes and autophagy. Finally, based on previous and current studies, the roles of NET formation and the potential therapeutic targets and strategies related to NETs in several well-studied types of cancers, including breast, lung, colorectal, pancreatic, blood, neurological, and cutaneous cancers, are separately reviewed and discussed.

## Introduction

Neutrophils are recognized as the most abundant leukocytes in the blood, comprising approximately 50%–70% of all circulating leukocytes in humans and 10%–25% in mice (1–3). They are widely acknowledged as vital members of both innate and adaptive immune responses and defendants against exogenous invaders, including various kinds of bacteria, viruses, and fungi (4–6). Under microbial infection or foreign invasion, neutrophils are rapidly activated and accumulated, which contribute to the restriction and clearance by triggering reactive oxygen species (ROS) production, endocytosis, degranulation, etc. (7). In 2004, Brinkmann et al. observed a special form of neutrophil degranulation, consisting of DNA fibers decorated with granule proteins, which were initially termed neutrophil extracellular traps (NETs) (8). They revealed that the formation of extracellular DNA traps contributed to the constraining and killing of invasive bacteria (8). In the last 17 years since their initial discovery and definition, numerous studies have been devoted to uncovering the characteristics of NETs. Studies have also demonstrated the physiological and pathological functions of self-defensive mechanisms in various types of disorders *via* the involvement of inflammatory and immune responses (9–12). Recently, NETs have been demonstrated to be involved in the pathogenesis and progression of malignant tumors. An increasing number of studies have revealed the pro-tumor effects of NETs. These effects are mediated *via* mechanisms including the establishment of an inflammatory microenvironment and interaction with other pro-tumor mechanisms such as inflammasomes and autophagy (13–17). In this review, knowledge on the biological characteristics of NETs is presented. Furthermore, the roles of NETs and potential therapeutic targets and strategies related to NETs in several types of cancer, including breast, lung, colorectal, pancreatic, blood, neurological, and cutaneous cancers, is discussed in detail through a review of the latest related studies.

## Part I: Biological Characteristics of NETs

NETs are extracellular strands of decondensed (unwound) DNA fibers in complex with histones and neutrophil granule proteins, including matrix metalloproteinase (MMP), neutrophil elastase (NE), myeloperoxidase (MPO), cathepsin G, complement factors, and other enzymatically active proteases and peptides (18–20). In the first few years since its initial report in 2004 (8), the term “neutrophil extracellular trap-osis (NETosis)” was widely used in related studies instead of NETs. The extensive use of the term NETosis was based on reports demonstrating that most extrusion of DNA strands resulted in their death, which allowed neutrophils to serve in immune reactions after their death (21–24). However, a strong concern was raised, since an increasing number of studies had reported that the occurrence of NETs does not necessarily lead to neutrophil death (9, 25–27). Therefore, it was strongly recommended by the Nomenclature Committee on Cell Death (NCCD) in 2018 that the term “NETosis” should be replaced with “NETs” or “NET formation” to include the DNA extrusion in the absence of cell death (28). Given this consideration, in this review, “NETs” or “NET formation” is used instead of “NETosis” in the following sections.

**Figure 1:**
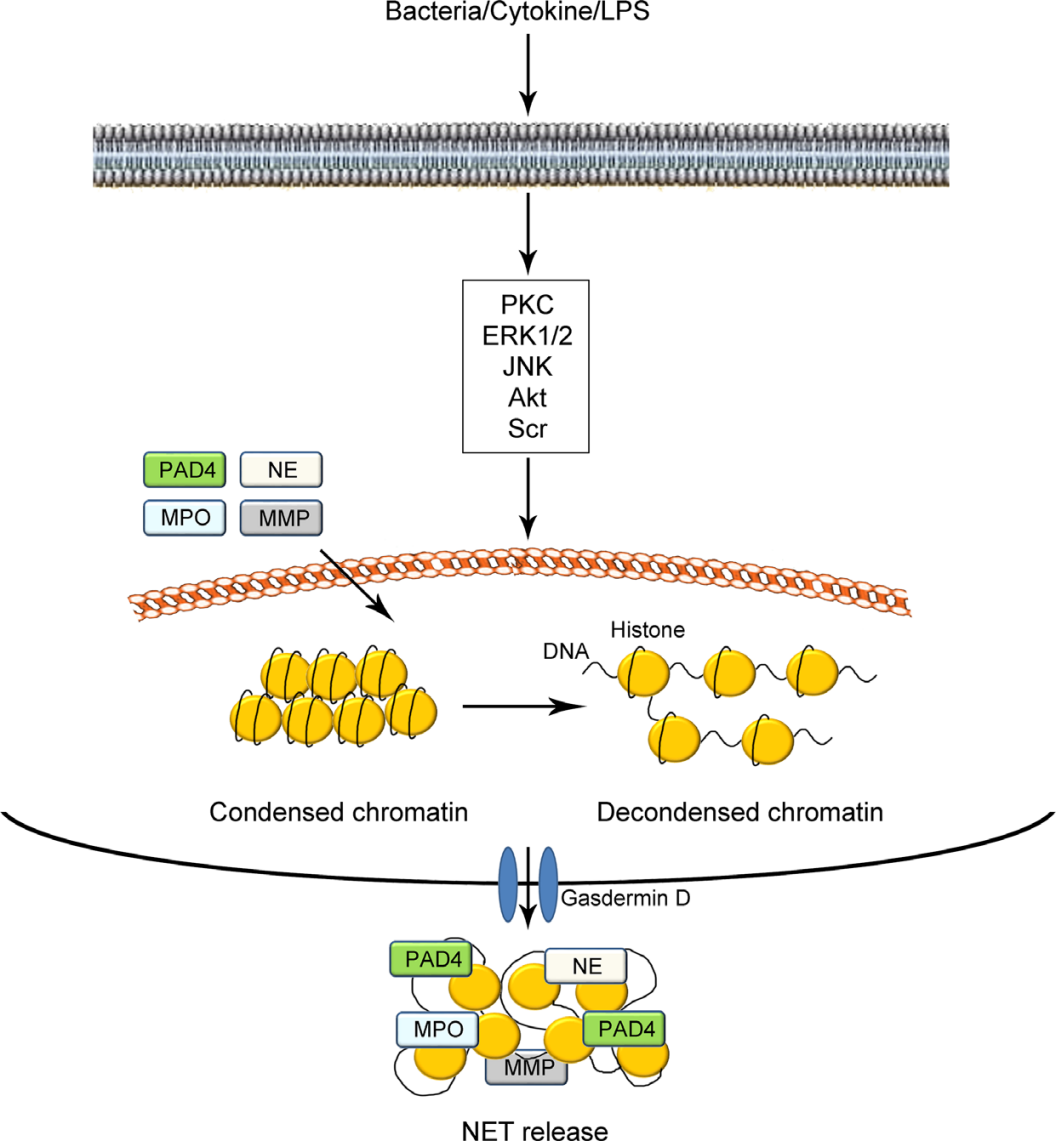
Schematic illustration of NET formation. Under certain stimuli such as bacterial infection, cytokines, and LPS challenge, neutrophils are activated *via the* modulation of cellular signaling pathways mediated by PKC, EK1/2, JNK, Akt, Scr, and so on. The tight electrostatic binding between DNA strands and histones in nucleosomes is weakened, mediated by two kinds of proteases including PAD4 and NE. The decondensed DNA with citrullinated histones catalyzed by PAD4 are decorated with several granule proteins including PAD4, NE, MPO, MMP, and gasdermin D and expelled from neutrophils as NETs. NETs, neutrophil extracellular traps; LPS, lipopolysaccharide; PKC, protein kinase C; ERK1/2, extracellular regulated protein kinase 1/2; JNK, c-Jun N-terminal kinase; PAD4, peptidyl arginine deiminase 4; MMP, matrix metalloproteinase; NE, neutrophil elastase; MPO, myeloperoxidase.

Under normal conditions, most DNA strands in neutrophils are highly wrapped around histones into heterochromatin within the nucleus (18, 29, 30). The protein–DNA interactions largely constrain the potential energy of DNA to extend, which leads to transcriptional inactivity (31). Under certain stimuli, such as microbial and sterile agents *in vivo* or phorbol 12-myristate 13-acetate (PMA), lipopolysaccharide (LPS), and intracellular calcium ion flux *in vitro*, the condensed DNA strands in neutrophils are uncoiled as fibrous polymers. The decondensation of DNA strands leads to the release of such potential energy, thus facilitating the formation of NETs (illustrated in **Figure 1**) (32, 33). To date, two proteases have been commonly acknowledged to be vital in the process of NET formation (18, 34–36). The first is peptidyl arginine deiminase 4 (PAD4), which catalyzes the conversion of arginine in histones to citrullines. Such citrullination significantly weakens the original positive charge of histones and weakens the strong histone-DNA binding, which leads to the decondensation of nuclear DNA and/or mitochondrial DNA. Besides PAD4, the other vital protease is NE, which is considered to facilitate the destruction of histone–DNA binding by cleaving histones. Deficiency in either PAD4 or NE in mice has been shown to prevent generation of NETs (37–39). After the decondensation of chromatins and disintegration of nuclei, DNA structures decorated with histones and granule proteins are extruded throughout the cellular membrane with the assistance of gasdermin D. Gasdermin D has been shown to function in the formation of pores, which results in the release of NETs (40, 41). However, several studies have demonstrated that PAD4 is not always necessary for NET formation. For instance, some researchers have argued that the role of PAD4 in NADPH-oxidase (NOX)-dependent NETs remains controversial. They observed the formation of NETs in the absence of detectable histone deamination (32, 42, 43). These studies indicate the complexity of the processes and mechanisms of NET formation.

To date, various signaling pathways have been shown to be involved in NET formation. Changes in the levels of signaling pathway-related proteins have been detected, including protein kinase C (PKC), extracellular regulated protein kinase 1/2 (ERK1/2), c-Jun N-terminal kinase (JNK), Akt, and Scr (44–46). In addition, several mechanisms, including those involved in inflammasomes and autophagy, have been shown to interact with NETs in diseases (47–49). Since the initial discovery of bacterial infection as a stimulating factor for NETs, an increasing number of stimuli have been uncovered in the recent decade to trigger NET formation. In addition to bacteria, several other factors, including interleukin (IL)-8 (a major neutrophil chemoattractant) (50, 51), PMA (a PKC activator) (52–54), LPS (a component of gram-negative bacteria) (55, 56), certain kinds of toxins, intracellular calcium ion flux, ionomycin, and A23187 (18, 42) have been shown to stimulate NET formation. It is notable that the mechanisms for the stimulation of NET formation differ between *in vitro* and *in vivo* conditions. For instance, LPS has been demonstrated to be effective in inducing NET formation *in vitro* through direct stimulation of neutrophils. However, it has been shown that LPS can only induce NET generation by binding activated platelets (thrombocytes) *in vivo* (57). Due to the complexity of *in vivo* conditions and the relative limitations of research techniques, a limited number of studies have addressed the specific inducers of NET formation *in vivo* (33). To date, several factors, including exogenous infections such as bacteria and fungi (10, 58, 59), inflammatory cytokine stimulation (60, 61) and interaction with activated platelets (62) have been recognized as stimuli *in vitro*. However, few specific agents have been proven to be effective in inducing NET formation *in vivo*.

Based on the knowledge of the biological and morphological characteristics of NETs, several methods have been developed for the detection and monitoring of NET formation, including immunofluorescence, immunohistochemistry, intravital microscopy, live cell imaging, DNA-intercalating dying techniques, and immunoblotting. These methods mainly target NET-related proteins, such as PAD4, NE, MPO, and MMP (33, 63–65). By means of these techniques and methods for NET detection, NETs have been demonstrated to be highly involved in the pathogenesis and progression of several kinds of disorders, including inflammatory bowel disease (66–68), multiple sclerosis (69, 70), atherosclerosis (71, 72), ischemic stroke (73), and some other autoimmune diseases (11, 20, 74). Moreover, an increasing number of studies have uncovered the involvement of NETs in the onset and development of malignant tumors, which will be discussed in detail in the following sections.

## Part II: NETs in Cancer

### General Ideas on the Relations Between NETs and Cancer

Notably, a search on the database of PubMed (www.ncbi.nlm.nih.gov/pubmed/) using the keywords “neutrophil extracellular traps” and (“cancer” or “tumor”) yielded 672 results by June 2021, with studies published in the last 4 years (2018–2021) accounting for 65.9% (443 in 672). Demers et al. (75) first reported the role of NETs in cancer. Since then, the prevailing view is that NETs produce a pro-tumor effect through the promotion of cancer cell proliferation, differentiation, metastasis, and other pathological characteristics in various types of malignant tumors (13, 76–81). For instance, it was demonstrated that NE, an important granule protein in NET microvesicles, could degrade the extracellular matrix and induce the phosphatidylinositol-4,5-bisphosphate 3-kinase (PI3K) pathway in cancer cells. The induction of the PI3K signaling pathway promotes cancer cell proliferation and migration (76, 77). Another member of the granule proteins, MMP, was also reported to promote tumor growth and metastasis through proteolysis of the extracellular matrix (13, 78). In addition, it has been revealed that the proteases in NETs can induce the remodeling of laminin, which triggers the integrin signaling pathway in cancer cells and the awakening of dormant tumor growth (13). Moreover, Teijeira et al. demonstrated that CXCR1 and CXCR2 agonist-induced NETs could wrap and coat cancer cells, which shielded them from clearance and the cytotoxic effects of cytolytic cytotoxic T lymphocytes (CTLs) and natural killer (NK) cells (79). These data indicate that NETs may function as a physical barrier preventing the interaction between cancer cells and surrounding inflammatory and immune populations (79, 80). It was further revealed that the levels of intratumoral NETs and supranormal preoperative serum MPO-DNA, regarded as a NET marker, were significantly increased in metastatic cancer tissues (81). They showed that NETs could facilitate the growth of stressed cancer cells by altering their bioenergetics, while the inhibition of NETs leads to cancer cell death (81).

Cancer-associated platelet activation has been shown to facilitate tumor progression and metastasis through protecting cancer cells from shear forces and assault of immune cells, opening the capillary endothelium to induce the epithelial–mesenchymal transition (EMT) program and secreting pro-tumor growth factors, etc. (82–84). Patients with malignant tumors demonstrate an increase in platelet activation (82). NETs have been shown to contribute to the formation of arterial, venous, and cancer-associated thrombosis (15, 85). These findings suggest that targeting NETs may serve as a potential and promising approach to reduce thrombosis and limit tumor progression and metastasis.

In addition, NETs have also been regarded as an important member of the dynamic tumor immune microenvironment (TIME), which may contribute greatly to impeding metastatic dissemination (51, 86). Several factors have been revealed to contribute to the formation of the TIME. Among them, cancer-associated fibroblasts (CAFs) are regarded as one of the most vital pro-tumor factors. Regarding the influence of NETs on CAFs, it was reported that the formation of NETs originated from CAFs in pancreatic ductal adenocarcinoma, thus producing a pro-tumor microenvironment (87). For the mechanism by which NETs influence the conversion of normal fibroblasts into CAFs in tumors, a nuclear factor (NF)-κB-dependent manner in stomal cells induced by activated neutrophils has been reported (88). Furthermore, the overwhelming production of fibroblast growth factors (FGFs) triggered by NETs in cancer was also shown to be a potential mechanism for the promotion of CAFs (89). However, due to the complexity of the conversion of normal fibroblasts to CAF, more research is required to explore the specific mechanisms mediated by NET regulation.

Although abundant experimental data suggest the pro-tumor effect of NETs on cancer, it is not in the absence of controversy (90, 91). Some researchers have argued that baseline NETs could produce an anti-tumor effect by directly killing cancer cells or limiting tumor growth and metastasis *via* stimulation of the immune system (90). In addition, NETs have been shown to limit the growth of cancer-related intestinal microbiota populations, thus inhibiting the proliferation and metastasis of colorectal cancer cells (91). Therefore, to ultimately take advantage of NETs for cancer treatment, more knowledge should be gained regarding the precise regulation of NETs.

Currently, there is still little knowledge on the specific mechanisms of NET formation in cancer. Although some findings have been obtained, there is no consensus regarding this issue. A previous study revealed that NETs were produced by neutrophils in a toll-like receptor 4 (TLR4) and high-mobility group box 1 (HMGB1)-dependent manner in lung cancer cells (92). In addition, it has also been reported that the chronically inflammatory microenvironment contributes to the formation of NETs *via* an MPO–NETs–antineutrophil antibody (ANCA) axis in cancer (93, 94). Researchers have shown that NETs might act as a central factor in the neutrophil–NET–cancer cycle, thus aggravating the pathogenesis and progression of cancer. Other researchers have revealed that NETs might be associated with other cellular processes, including inflammasomes and autophagy, which will be discussed in the following sections.

## Inflammasomes, NETs, and Cancer

Inflammasomes are multi-protein complexes that are widely regarded as important factors of inflammation and innate immunity responsible for the activation of inflammatory and immune responses through the recognition of pathogen-associated molecular patterns (PAMPs) or danger-associated molecular patterns (DAMPs) (95). To date, several types of inflammasomes have been reported, including the NOD-like receptor family, pyrin domain-containing 1 (NLRP1), NLRP2, NLRP3, NLR family caspase recruitment domain-containing protein 4 (NLRC4), and double-stranded DNA sensors absent in melanoma 2 (AIM2) (96–98). As previously reviewed by us, the activation of inflammasomes involves the cleavage of procaspase-1 into caspase-1, which subsequently catalyzes the production and secretion of inflammatory cytokines, including IL-1β and IL-18 (99, 100). The involvement of NF-κB signaling in the initiation of inflammasome activation leads to increased production of inflammasome components (99, 100). Recently, numerous studies conducted by our laboratory and others have demonstrated the roles of inflammasomes in various diseases, including inflammatory bowel disease, multiple sclerosis, atherosclerosis, stroke, and malignant tumors (96, 101–103). In cancer, it has been revealed that the over-induction of inflammasomes largely influences cancer cell death, proliferation, and even tumor growth microenvironment, such as intestinal microbiota populations and fibroblasts, which indicates the wealth of putative inflammasome-based targeted therapies for cancer (104–110).

As important components in immune reactions, NETs have been shown to be closely linked to inflammasomes in some autoimmune diseases and cardiovascular disorders (19, 111, 112). For instance, it has been previously described that the formation of NETs by neutrophils could trigger the synthesis of inflammasome-related IL-1β and IL-18 in macrophages through cathelicidin LL-37-mediated potassium efflux from the cells. The production of IL-1β and IL-18 subsequently promotes NET formation during the occurrence of cardiovascular diseases (19, 113). Similar crosstalk between inflammasomes and NETs has been reported in cancer. It has been demonstrated that NET-associated serine proteases such as NE could act as alternative enzymes for processing inflammasome-related IL-1β and IL-18, which subsequently leads to the modulation of PGRN inactivation and MMP-9 activation in cancer (114). In addition, Albrengues et al. (13) reported in lung cancer that NETs and NETs-mediated extracellular matrix remodeling acted as critical mediators of awakening of dormant cancer cells by LPS-mediated formation of inflammasomes in mice. This process is mediated by the binding between NET-DNA and extracellular matrix protein laminin and bringing NE and MMP-9 to their substrates (13). To date, little evidence has been available regarding the crosstalk between inflammasomes and NETs in cancer (13, 114, 115). However, future studies should further elucidate such interactions as they may unravel potential novel therapeutic strategies for the treatment of cancer.

### Autophagy, NETs, and Cancer

Autophagy, commonly recognized as a vital metabolic mechanism relying on lysosomes, functions in degrading and recycling long-lived, misfolded proteins and damaged organelles to maintain cellular homeostasis (116–118). As previously described, under organic stress, such as nutrient deprivation or inflammatory loading, cytoplasmic materials are targeted and sequestrated into autophagosomes, which are regarded as the functional units of autophagy (119–121). The autophagosomes fuse with lysosomes to form autolysosomes (119–121). Notably, Yoshimori Ohsumi was awarded the 2016 Nobel Prize in Medicine or Physiology for exploring the cellular autophagy processes (122). Since its discovery by Christian de Duve in the 1960s (123), numerous studies from our laboratory and others have uncovered the involvement of autophagy in various diseases, including cardiovascular disorders, autoimmune diseases, metabolic abnormalities, malignant tumors, neurodegenerative diseases, and gastrointestinal diseases (124–129).

The influence of autophagy on the pathogenesis and progression of cancer tends to be regarded as a double-edged sword (130–134). In contrast, autophagy has been considered to maintain stemness, induce recurrence, and develop resistance of cancer cells to anticancer agents. The administration of rapamycin, an autophagy inducer, has been shown to be effective in alleviating cancer (131–133). Additionally, autophagy has also been reported to inhibit tumor initiation through the induction of autophagic cell death, and chloroquine, an autophagy inhibitor, has been used in anti-tumor therapy (131, 134). These studies indicate the complexity of autophagy mechanisms in cancer.

The dichotomous effects and complex mechanisms of interactions between autophagy and NETs have also been demonstrated in cancer. It has been reported that the induction of autophagy (formation of autolysosomes) in leukemia cells leads to the release of NETs, which results in the deterioration of acute promyelocytic leukemia (135). In addition, Boone et al. (136) revealed that NETs were upregulated in pancreatic cancer *via* receptor for advanced glycation end products (RAGE)-dependent/autophagy-mediated pathways. Regarding the effect of autophagy and NET crosstalk in cancer, it was previously revealed that the administration of chloroquine significantly reduced the hypercoagulability in pancreatic cancer by inhibiting NETs, suggesting a positive effect of suppressing autophagy–NET interaction in the alleviation of cancer (137). However, autophagy has been reported to be involved in the effects of interferon (IFN)-γ on cell growth inhibition and cytotoxicity in lung epithelial malignancies *via* the induction of PAD4-mediated NETs (138). Consequently, to ultimately take advantage of interactions between autophagy and NETs in cancer treatment, further studies are required to explore this issue.

## Part III: NETs in Different Types of Cancer

During the 16 years since the initial discovery of NETs in 2004, an increasing number of researchers have focused on the study of NETs in cancer. Fortunately, to date, the effects of NETs in several popular types of cancer have been widely revealed to illustrate the whole picture. In this section, the roles of NETs and potential therapeutic targets and strategies related to NETs in several types of cancers, including breast, lung, colorectal, pancreatic, blood, neurological, and cutaneous cancers are described and discussed in detail based on the latest studies available in the current database (listed in **Table 1**).

**Table 1:** Potential pathophysiological and molecular mechanisms of NETs on different kinds of cancers.

Cancer	Pathophysiological AND molecular mechanisms	Reference
BreAst cancer	In correspondence with cancer-associated thrombosis occurrence in lung	(75)
	Release of cancer extracellular chromatin networks (CECN)	(139)
	Activating the epithelial–mesenchymal transition (EMT) program	(140)
	Acting as a chemotactic factor in assistant with a transmembrane protein CCDC25	(141)
	Interaction with inflammasomes	(142)
Lung cancer	Activating integrin α3β1 signaling *via* NE/MMP 9-induced cleavage of laminin	(13)
	Induced by a danger-associated molecular pattern protein high-mobility group box 1 (HMGB1)	(92)
	Induced by extracellular RNAs produced by lung cancer cells	(143)
	Preventing hypercoagulation and lung carcinogenesis	(144)
Colorectal cancer	Inducing procoagulant activity (PCA) and leading to a close interaction with platelets and endothelial cells	(145)
	NETs-associated carcinoembryonic Ag cell adhesion molecule 1 (CEACAM1) acted as an essential element for the interaction between NETs and colorectal cancer cells	(146)
	Mediated by KRAS mutation transferred by exosomes	(147)
	Forming a positive loop connecting between NETs and colorectal cancer liver metastasis mediated by IL-8	(148)
Pancreatic cancer	Activating cancer-associated fibroblasts	(87)
	Modulating cell–cell junctions	(149)
	Epithelial–mesenchymal transition *via* IL-1β/epidermal growth factor receptor (EGFR)/extracellular regulated protein kinase (ERK) pathway	(150)
	Stimulating platelets and release of tissue factor *via* autophagy	(137)
Blood cancerS	Stimulating citrullination of histone H3 *via the* activation of PAD4 in multiple myeloma	(151)
	Activating platelets in myeloproliferative neoplasms	(152)
	Mediated by the Janus kinase (JAK)-activator of transcription (STAT) signaling	(153)
	Inhibited by ibrutinib in chronic lymphocytic leukemia	(154)
Neurological cancers	Involved by highly sensitive Troponin T (hsTnT)	(155)
	Regulated by the high-mobility group box 1 (HMGB1)/advanced glycation end products (RAGE)/IL-8 axis	(156)
Cutaneous cancers	Promoting the spontaneous and immunotherapy-induced adverse reaction of melanoma	(157)
	Association of IL-8 and NETs	(158)
	Producing an anti-tumor polarization of tumor-associated neutrophils mediated by type I interferons (IFNs)-related NETs suppression	(159)

### Breast Cancer

Breast cancer is regarded as one of the three most commonly diagnosed cancers worldwide, especially in women (160). Breast cancer is one of the most studied type of cancer-related to NETs. It was first reported by Demers et al. (75) that, in a murine late-stage breast cancer model, the formation of NETs corresponded with cancer-associated thrombosis in the lung. The formation of thrombosis contributed to a poor prognosis and cancer-caused death. In addition, the expression levels of PAD4 genes were shown to be high in murine breast cancer 4T1 cells and PAD4-mediated NETs, which contributed to the release of cancer extracellular chromatin networks (CECN) both *in vitro* and *in vivo* (139). PAD4-mediated NETs were demonstrated to promote breast tumor growth and cancer metastasis into the lung, since the deletion of PAD4 genes in mouse models largely attenuated breast cancer cell proliferation and migration (139). In addition, Martins-Cardoso et al. (140) revealed that NETs promoted a pro-metastatic phenotype in human breast cancer cells by inducing the EMT program.

Regarding the exploration of therapeutic targets related to NETs in the treatment of breast cancer, a brilliant study by Yang et al. (141) uncovered a potential specific mechanism for the influence of NETs in breast cancer metastasis. In this study, the researchers revealed that the DNA components of NETs (NET-DNA) could act as a chemotactic factor to attract breast cancer cells rather than merely “trap” them, thus leading to the occurrence of liver metastases in patients with early-stage breast cancer (141). They also suggested that the transmembrane protein CCDC25 might act as a potential NET-DNA receptor in breast cancer cells by sensing extracellular DNA. The activation of CCDC25 consequently enhanced cell motility through the subsequent activation of the ILK-β-parvin pathway. These data indicate an appealing therapeutic strategy that takes advantage of targeting CCDC25 for cancer metastasis prevention (141). It has been commonly revealed that patients with breast cancer are at a relatively higher risk of developing thrombosis. Gomes et al. (142) demonstrated that blockade of inflammasome-related IL-1β production and secretion attenuated cancer-associated thrombosis in a NETs-dependent breast cancer model, indicating the vital role of the crosstalk between NETs and inflammasomes in the treatment of breast cancer.

### Lung Cancer

Lung cancer is the most diagnosed and frequent cause of cancer-related deaths worldwide, with approximately 1.8 million newly diagnosed patients and 1.6 million deaths each year (161). Thus far, NETs have been demonstrated to modulate the biological characteristics of lung cancer cells, thus influencing the pathogenesis and progression of lung carcinoma. For instance, a brilliant study published in *Science* revealed that sustained lung inflammation mediated by smoke exposure or nasal instillation of LPS could awaken dormant cancer cells and facilitate metastasis through the induction of NET formation (13). Mechanistic analysis revealed that this process was potentially *via* NE/MMP-9-induced cleavage of laminin, which subsequently led to the activation of integrin α3β1 signaling in dormant cancer cells (13). However, it is worth mentioning the difference between lung cancer patients and tumor-bearing mice models pointed out by Arpinati et al. (162). They argued in a recent study that no predisposition of neutrophils to release NETs in patients with lung cancer was found to be similar to that in mice compared with healthy controls. These results indicate that attention should be paid to the translation of experimental results obtained from animal studies into clinical applications (162).

Regarding therapeutic targets related to NETs in lung cancer, it was previously revealed that the DAMP protein HMGB1 released by lung cancer cells contributes to the induction of NETs that was dependent on the activation of TLR4 (92). In addition, extracellular RNAs (exRNAs) produced by lung cancer cells have also been demonstrated to induce NET formation, which promotes cancer cell proliferation and migration (143). In addition to exploring therapeutic targets, some researchers have focused on the development of therapeutic strategies for the treatment of lung cancer-related to NETs, using certain natural compounds. Recently, Li et al. (144) revealed that emodin, the main bioactive component of *Rheum palmatum*, could significantly prevent hypercoagulation and lung carcinogenesis by suppressing NET formation in lung cancer animal models. However, most related research is still in the stage of animal models. Therefore, more clinical studies are required for the development of cancer therapies based on NETs.

### Colorectal Cancer

According to the analyzed data in 2019, colorectal cancer is considered to be the fourth most deadly cancer worldwide, with 900,000 deaths annually (163). Aging, western lifestyle, and bowel inflammatory conditions (e.g., inflammatory bowel diseases) serve as risk factors for the oncogenesis of colorectal cancer (163, 164). Richardson et al. (165) demonstrated that patients with colorectal cancer have significantly increased NET formation compared with healthy volunteers, and high levels of NETs were shown to be associated with adverse patient outcomes. Furthermore, NETs have been reported to be involved in venous thrombogenesis in patients with colorectal cancer *via* the induction of procoagulant activity (PCA), which led to a close interaction with platelets and endothelial cells (145). These data indicate that targeting NETs may be a potential and promising way to tackle thrombosis in colorectal cancer.

Exploring potential therapeutic targets related to NETs in colorectal cancer, Rayes et al. (146) revealed that NETs-associated carcinoembryonic antigen cell adhesion molecule 1 (CEACAM1) acts as an essential element for the interaction between NETs and colorectal cancer cells, and deficiency in CEACAM1 significant decreases cancer cell adhesion, migration, and metastasis. Regarding the induction of NET formation, *KRAS* mutation has been shown to contribute to neutrophil recruitment and NET formation through exosomes in colorectal cancer, which uncovered a novel mechanism of regulation of NET formation in colorectal cancer (147). In addition, IL-8, a tumor inflammatory cytokine, has been reported to be part of a positive loop connecting NETs and colorectal cancer liver metastasis (148). However, further studies are required to develop effective therapeutic strategies for colorectal cancer, taking advantage of these targets.

### Pancreatic Cancer

Pancreatic cancer is a highly lethal cancer in the absence of a standard screening program since most patients remain asymptomatic until they reach an advanced stage (166). A clinical study demonstrated that tumor-infiltrating neutrophils (TINs) and their generated NETs could be treated as prognostic factors for pancreatic cancer independent of the TNM staging system (167). NETs have also been shown to promote liver micrometastasis in pancreatic cancer by activating CAFs (87). Notably, it has been commonly recognized that pancreatic cancer induces a hypercoagulable state, which could result in clinically apparent thrombosis (168). An increasing number of studies have demonstrated the influence of NETs on hypercoagulability and thrombogenesis (137, 149, 168). For instance, Yu et al. (149) showed an association between the upregulation of NETs and PCA in patients with pancreatic cancer through the modulation of cell–cell junctions. Furthermore, Hisada et al. (169) revealed that neutrophils and NETs contribute to venous thrombosis in mice bearing human pancreatic tumors and patients with pancreatic cancer, indicating a thrombogenic effect of NETs in pancreatic cancer.

Regarding therapeutic targets related to NETs in pancreatic cancer, Jin et al. (150) revealed that NETs could promote migration and invasion of pancreatic cancer cells *via* EMT and the IL-1β/epidermal growth factor receptor (EGFR)/ERK pathway. These results indicate that blockade of the IL-1β/EGFR/ERK signaling pathway might serve as a potential strategy for the alleviation of pancreatic cancer. In addition, it has been reported that targeting the interaction between NETs and autophagy reduces hypercoagulability in pancreatic cancer (137). They showed that the administration of chloroquine for the blockade of autophagy significantly lowered the rates of venous thromboembolism in patients with pancreatic cancer by suppressing autophagy-related NET formation. However, further studies are needed for successful clinical application.

### Blood Cancers

Blood cancers are a group of “invisible” cancers, as tumors are seldomly observed in blood cancers (170). In a broad sense, blood cancers include leukemia, multiple myeloma, and malignant lymphoma. Regarding the effects of NETs on blood cancers, a statistically significant increase in NET levels was found in patients with multiple myeloma (MM) compared to healthy volunteers both in the serum and plasma (171). Furthermore, MM cells have been shown to stimulate citrullination of histone H3 and subsequently lead to the formation of NETs through the activation of PAD4 (151). In addition, NETs have been reported to contribute to thrombogenesis, a major cause of mortality in patients with myeloproliferative neoplasms (MPNs) *via* platelet activation (152).

Regarding the therapeutic targets related to NETs in blood cancers, it was reported by Wolach et al. (153) that NET formation was increased in mice MPN models *via* the Janus kinase (JAK)-activator of transcription (STAT) signaling. The administration of ruxolitinib, a JAK2 inhibitor, effectively suppressed NET formation and reduced thrombosis. In addition, NET formation has been previously shown to contribute to the pathogenesis of chronic lymphocytic leukemia (CLL), and ibrutinib, a widely used chemotherapeutic drug, alleviated CLL by slightly impairing NET production in patients with CLL (154). Based on these findings, the modulation of NET formation may be a potential and promising therapeutic strategy for the treatment of blood cancers.

### Neurological Cancers

Neurological cancers are defined as a group of primary or metastatic cancers of the central nervous system (CNS) (172). According to the World Health Organization (WTO) in 2016 (173), neurological cancers comprise diffuse astrocytic and oligodendroglial tumors, other astrocytic tumors, ependymal tumors, other gliomas, lymphomas of the CNS, and metastatic cancers. Regarding the role of NETs in neurological cancers, it was previously reported that NETs could significantly promote cancer-associated arterial microthrombosis in cancer patients presenting with ischemic stroke and large elevations of highly sensitive troponin T (hs-TnT) (155). In addition, in patients with high-grade gliomas, a high level of thrombogenic NETs produced by neutrophils was detected, which resulted in an increase in venous thromboembolism (VTE) (174).

Regarding therapeutic targets related to NETs in neurological cancers, Zha et al. (156) revealed that NETs generated by TINs mediated the crosstalk between glioma and tumor microenvironment in patients with malignant glioma. These effects were demonstrated to be regulated by the HMGB1/RAGE/IL-8 axis. However, little is known about the specific mechanisms of NET formation in neurological cancers. Consequently, further studies are required to ultimately develop therapies targeting NETs.

### Cutaneous Cancers

Cutaneous cancers, also known as “skin cancers”, are a group of cancers that occur in the skin tissue. Cutaneous cancers are divided into melanoma and non-melanoma, with the latter including basal cell carcinoma and squamous cell carcinoma (175). The mechanisms underlying the pathogenesis and progression of cutaneous cancers are complex and remain unclear. To date, modern research has demonstrated that NETs are closely related to cutaneous cancers. NETs have been reported to promote inflammation-mediated skin tumor cell growth in mouse models (176). NETs have also been shown to contribute to spontaneous and immunotherapy-induced adverse reactions of melanoma murine models (157). In addition, similar to other tissues, NET-mediated thrombosis was also found in the skin tissue, indicating the positive effects of NETs on thrombosis in cutaneous disorders (177).

IL-8 has been reported to be a potential therapeutic target related to NETs, since the association of IL-8 and NETs was revealed in patients with metastatic melanoma (158). In addition, type I IFNs, widely used anti-inflammatory agents, have been shown to induce anti-tumor polarization of tumor-associated neutrophils in murine models and melanoma patients. The transfer of neutrophils into an anti-tumor disturbs the formation of NETs and thus produces a tumor-suppressive effect (159). However, additional studies are needed to develop therapies for cutaneous cancers that target NETs.

## Conclusion

**Figure 2:**
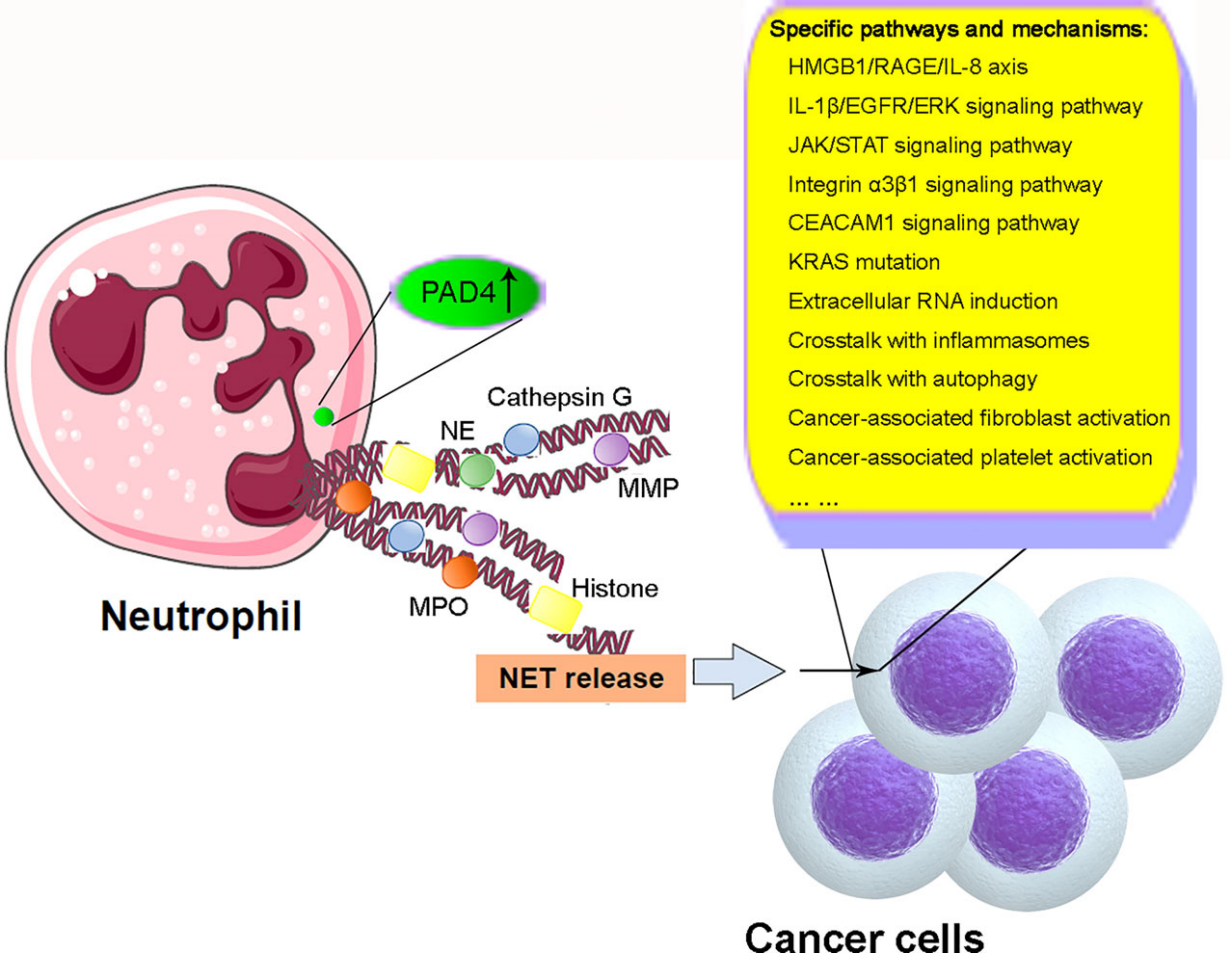
Schematic illustration of mechanism of NETs in cancer. Under cancer-related stimulation, the level and function of PAD4 are enhanced, leading to the citrullination of histones and subsequent decondensation of chromatins. NET microvesicles with DNA-structural fibers decorated by histones, MMP, NE, MPO, cathepsin G, and other granule proteins are extruded from neutrophils. NET formation modulates the biological characteristics of cancer cells in proliferation, differentiation, migration, and metastasis through the involvement and crosstalk with some specific pathways and mechanisms including HMGB1/RAGE/IL-8 axis, IL-1β/EGFR/ERK signaling pathway, JAK/STAT signaling pathway, integrin α3β1 signaling pathway, CEACAM1 signaling pathway, KRAS mutation, extracellular RNA induction and crosstalk with inflammasomes and autophagy, and cancer-associated fibroblast and platelet activation. NETs, neutrophil extracellular traps; PAD4, peptidyl arginine deiminase 4; MMP, matrix metalloproteinase; NE, neutrophil elastase; MPO, myeloperoxidase; HMGB1, high-mobility group box 1; RAGE, advanced glycation end products; EGFR, epidermal growth factor receptor; ERK, extracellular regulated protein kinase; JAK, Janus kinase; STAT, activator of transcription; CEACAM1, carcinoembryonic Ag cell adhesion molecule 1.

Overall, recent studies have demonstrated the important roles of NETs in cancer through modulation of the biological characteristics of cancer cells including proliferation, differentiation, and metastasis and induction of cancer-related thrombogenesis (illustrated in **Figure 2**). So far, we have gained significant knowledge on the biological features and regulation of NET formation, as well as the mechanisms of the effects of NETs on different types of cancers. However, because of the limitations of current studies, the specific mechanisms of NETs and the crosstalk between NETs and other cancer-related processes, including inflammasomes and autophagy, remain unclear. The specific mechanisms underlying NET formation also remain unclear. Therefore, additional studies are required for the successful application of knowledge regarding NET formation and function in clinical practice through the development of novel and promising therapeutic strategies against cancer.

## Author Contributions

B-ZS and YY retrieved concerned literatures and wrote the manuscript. J-PL designed the table and figures. E-QL and N-LC revised the manuscript. All authors contributed to the article and approved the submitted version.

## Funding

This work was supported by a grant from Ministry of Science and Technology of the People’s Republic of China (No. 2016YFC1303600) and grants from General Hospital of the Chinese People’s Liberation Army (No. 2018ZD-006 and 2018MS-012).

## Conflict of Interest

The authors declare that the research was conducted in the absence of any commercial or financial relationships that could be construed as a potential conflict of interest.

## Publisher’s Note

All claims expressed in this article are solely those of the authors and do not necessarily represent those of their affiliated organizations, or those of the publisher, the editors and the reviewers. Any product that may be evaluated in this article, or claim that may be made by its manufacturer, is not guaranteed or endorsed by the publisher.

## References

[1] Rosales C. Neutrophils at the Crossroads of Innate and Adaptive Immunity. J Leukoc Biol (2020) 108:377–96. 10.1002/JLB.4MIR0220-574RR 32202340

[2] He R Chen Y Cai Q. The Role of the LTB4-BLT1 Axis in Health and Disease. Pharmacol Res (2020) 158:104857. 10.1016/j.phrs.2020.104857 32439596

[3] Mestas J Hughes CC. Of Mice and Not Men: Differences Between Mouse and Human Immunology. J Immunol (2004) 172:2731–8. 10.4049/jimmunol.172.5.2731 14978070

[4] Brinkmann V Zychlinsky A. Neutrophil Extracellular Traps: Is Immunity the Second Function of Chromatin? J Cell Biol (2012) 198:773–83. 10.1083/jcb.201203170 PMC3432757 22945932

[5] Bianchi M Hakkim A Brinkmann V Siler U Seger RA Zychlinsky A. Restoration of NET Formation by Gene Therapy in CGD Controls Aspergillosis. Blood (2009) 114:2619–22. 10.1182/blood-2009-05-221606 PMC2756123 19541821

[6] Branzk N Lubojemska A Hardison SE Wang Q Gutierrez MG Brown GD. Neutrophils Sense Microbe Size and Selectively Release Neutrophil Extracellular Traps in Response to Large Pathogens. Nat Immunol (2014) 15:1017–25. 10.1038/ni.2987 PMC4236687 25217981

[7] Mayadas TN Cullere X Lowell CA. The Multifaceted Functions of Neutrophils. Annu Rev Pathol (2014) 9:181–218. 10.1146/annurev-pathol-020712-164023 24050624 PMC4277181

[8] Brinkmann V Reichard U Goosmann C Fauler B Uhlemann Y Weiss DS. Neutrophil Extracellular Traps Kill Bacteria. Science (2004) 303:1532–5. 10.1126/science.1092385 15001782

[9] Papayannopoulos V. Neutrophil Extracellular Traps in Immunity and Disease. Nat Rev Immunol (2018) 18:134–47. 10.1038/nri.2017.105 28990587

[10] Sollberger G Tilley DO Zychlinsky A. Neutrophil Extracellular Traps: The Biology of Chromatin Externalization. Dev Cell (2018) 44:542–53. 10.1016/j.devcel.2018.01.019 29533770

[11] Lee KH Kronbichler A Park DD Park Y Moon H Kim H. Neutrophil Extracellular Traps (NETs) in Autoimmune Diseases: A Comprehensive Review. Autoimmun Rev (2017) 16:1160–73. 10.1016/j.autrev.2017.09.012 28899799

[12] Brinkmann V. Neutrophil Extracellular Traps in the Second Decade. J Innate Immun (2018) 10:414–21. 10.1159/000489829 PMC6784051 29909412

[13] Albrengues J Shields MA Ng D Park CG Ambrico A Poindexter ME. Neutrophil Extracellular Traps Produced During Inflammation Awaken Dormant Cancer Cells in Mice. Science (2018) 361:eaao4227. 10.1126/science.aao4227 30262472 PMC6777850

[14] Park J Wysocki RW Amoozgar Z Maiorino L Fein MR Jorns J. Cancer Cells Induce Metastasis-Supporting Neutrophil Extracellular DNA Traps. Sci Transl Med (2016) 8:361ra138. 10.1126/scitranslmed.aag1711 PMC5550900 27798263

[15] Thalin C Hisada Y Lundstrom S Mackman N Wallen H. Neutrophil Extracellular Traps: Villains and Targets in Arterial, Venous, and Cancer-Associated Thrombosis. Arterioscler Thromb Vasc Biol (2019) 39:1724–38. 10.1161/ATVBAHA.119.312463 PMC6703916 31315434

[16] Jorch SK Kubes P. An Emerging Role for Neutrophil Extracellular Traps in Noninfectious Disease. Nat Med (2017) 23:279–87. 10.1038/nm.4294 28267716

[17] Jung HS Gu J Kim JE Nam Y Song JW Kim HK. Cancer Cell-Induced Neutrophil Extracellular Traps Promote Both Hypercoagulability and Cancer Progression. PLoS One (2019) 14:e0216055. 10.1371/journal.pone.0216055 31034495 PMC6488070

[18] Sorensen OE Borregaard N. Neutrophil Extracellular Traps - the Dark Side of Neutrophils. J Clin Invest (2016) 126:1612–20. 10.1172/JCI84538 PMC4855925 27135878

[19] Bonaventura A Vecchie A Abbate A Montecucco F. Neutrophil Extracellular Traps and Cardiovascular Diseases: An Update. Cells (2020) 9:231. 10.3390/cells9010231 PMC7016588 31963447

[20] Fousert E Toes R Desai J. Neutrophil Extracellular Traps (NETs) Take the Central Stage in Driving Autoimmune Responses. Cells (2020) 9:915. 10.3390/cells9040915 PMC7226846 32276504

[21] Yipp BG Petri B Salina D Jenne CN Scott BN Zbytnuik LD. Infection-Induced NETosis Is a Dynamic Process Involving Neutrophil Multitasking *In Vivo* . Nat Med (2012) 18:1386–93. 10.1038/nm.2847 PMC4529131 22922410

[22] Mesa MA Vasquez G. NETosis. Autoimmune Dis (2013) 2013:651497. 10.1155/2013/651497 23476749 PMC3576733

[23] Kaplan MJ Radic M. Neutrophil Extracellular Traps: Double-Edged Swords of Innate Immunity. J Immunol (2012) 189:2689–95. 10.4049/jimmunol.1201719 PMC3439169 22956760

[24] Gupta AK Joshi MB Philippova M Erne P Hasler P Hahn S. Activated Endothelial Cells Induce Neutrophil Extracellular Traps and Are Susceptible to NETosis-Mediated Cell Death. FEBS Lett (2010) 584:3193–7. 10.1016/j.febslet.2010.06.006 20541553

[25] Masuda S Nakazawa D Shida H Miyoshi A Kusunoki Y Tomaru U. NETosis Markers: Quest for Specific, Objective, and Quantitative Markers. Clin Chim Acta (2016) 459:89–93. 10.1016/j.cca.2016.05.029 27259468

[26] Yang H Biermann MH Brauner JM Liu Y Zhao Y Herrmann M. New Insights Into Neutrophil Extracellular Traps: Mechanisms of Formation and Role in Inflammation. Front Immunol (2016) 7:302. 10.3389/fimmu.2016.00302 27570525 PMC4981595

[27] White PC Chicca IJ Cooper PR Milward MR Chapple IL. Neutrophil Extracellular Traps in Periodontitis: A Web of Intrigue. J Dent Res (2016) 95:26–34. 10.1177/0022034515609097 26442948

[28] Galluzzi L Vitale I Aaronson SA Abrams JM Adam D Agostinis P. Molecular Mechanisms of Cell Death: Recommendations of the Nomenclature Committee on Cell Death 2018. Cell Death Differ (2018) 25:486–541. 10.1038/s41418-017-0012-4 29362479 PMC5864239

[29] Hamam HJ Khan MA Palaniyar N. Histone Acetylation Promotes Neutrophil Extracellular Trap Formation. Biomolecules (2019) 9:32. 10.3390/biom9010032 PMC6359456 30669408

[30] Tsourouktsoglou TD Warnatsch A Ioannou M Hoving D Wang Q Papayannopoulos V. Histones, DNA, and Citrullination Promote Neutrophil Extracellular Trap Inflammation by Regulating the Localization and Activation of TLR4. Cell Rep (2020) 31:107602. 10.1016/j.celrep.2020.107602 32375035

[31] Van HT Santos MA. Histone Modifications and the DNA Double-Strand Break Response. Cell Cycle (2018) 17:2399–410. 10.1080/15384101.2018.1542899 PMC6342081 30394812

[32] Kenny EF Herzig A Kruger R Muth A Mondal S Thompson PR. Diverse Stimuli Engage Different Neutrophil Extracellular Trap Pathways. Elife (2017) 6:e24437. 10.7554/eLife.24437 28574339 PMC5496738

[33] Boeltz S Amini P Anders HJ Andrade F Bilyy R Chatfield S. To NET or Not to NET:current Opinions and State of the Science Regarding the Formation of Neutrophil Extracellular Traps. Cell Death Differ (2019) 26:395–408. 10.1038/s41418-018-0261-x 30622307 PMC6370810

[34] Sur Chowdhury C Giaglis S Walker UA Buser A Hahn S Hasler P. Enhanced Neutrophil Extracellular Trap Generation in Rheumatoid Arthritis: Analysis of Underlying Signal Transduction Pathways and Potential Diagnostic Utility. Arthritis Res Ther (2014) 16:R122. 10.1186/ar4579 24928093 PMC4229860

[35] Hilscher MB Sehrawat T Arab JP Zeng Z Gao J Liu M. Mechanical Stretch Increases Expression of CXCL1 in Liver Sinusoidal Endothelial Cells to Recruit Neutrophils, Generate Sinusoidal Microthombi, and Promote Portal Hypertension. Gastroenterology (2019) 157:193–209.e9. 10.1053/j.gastro.2019.03.013 30872106 PMC6581607

[36] Diaz-Godinez C Fonseca Z Nequiz M Laclette JP Rosales C Carrero JC. Entamoeba Histolytica Trophozoites Induce a Rapid Non-Classical NETosis Mechanism Independent of NOX2-Derived Reactive Oxygen Species and PAD4 Activity. Front Cell Infect Microbiol (2018) 8:184. 10.3389/fcimb.2018.00184 29922599 PMC5996068

[37] Sorensen OE Clemmensen SN Dahl SL Ostergaard O Heegaard NH Glenthoj A. Papillon-Lefevre Syndrome Patient Reveals Species-Dependent Requirements for Neutrophil Defenses. J Clin Invest (2014) 124:4539–48. 10.1172/JCI76009 PMC4191054 25244098

[38] Rabadi M Kim M D’Agati V Lee HT. Peptidyl Arginine Deiminase-4-Deficient Mice Are Protected Against Kidney and Liver Injury After Renal Ischemia and Reperfusion. Am J Physiol Renal Physiol (2016) 311:F437–49. 10.1152/ajprenal.00254.2016 PMC5008675 27335376

[39] Raup-Konsavage WM Wang Y Wang WW Feliers D Ruan H Reeves WB. Neutrophil Peptidyl Arginine Deiminase-4 has a Pivotal Role in Ischemia/Reperfusion-Induced Acute Kidney Injury. Kidney Int (2018) 93:365–74. 10.1016/j.kint.2017.08.014 PMC5794573 29061334

[40] Sollberger G Choidas A Burn GL Habenberger P Di Lucrezia R Kordes S. Gasdermin D Plays a Vital Role in the Generation of Neutrophil Extracellular Traps. Sci Immunol (2018) 3:eaar6689. 10.1126/sciimmunol.aar6689 30143555

[41] Tall AR Westerterp M. Inflammasomes, Neutrophil Extracellular Traps, and Cholesterol. J Lipid Res (2019) 60:721–27. 10.1194/jlr.S091280 PMC6446695 30782961

[42] Ravindran M Khan MA Palaniyar N. Neutrophil Extracellular Trap Formation: Physiology, Pathology, and Pharmacology. Biomolecules (2019) 9:365. 10.3390/biom9080365 PMC6722781 31416173

[43] Douda DN Khan MA Grasemann H Palaniyar N. SK3 Channel and Mitochondrial ROS Mediate NADPH Oxidase-Independent NETosis Induced by Calcium Influx. Proc Natl Acad Sci U S A (2015) 112:2817–22. 10.1073/pnas.1414055112 PMC4352781 25730848

[44] DeSouza-Vieira T Guimaraes-Costa A Rochael NC Lira MN Nascimento MT Lima-Gomez PS. Neutrophil Extracellular Traps Release Induced by Leishmania: Role of PI3Kgamma, ERK, PI3Ksigma, PKC, and [Ca2+]. J Leukoc Biol (2016) 100:801–10. 10.1189/jlb.4A0615-261RR PMC5014744 27154356

[45] Fonseca Z Diaz-Godinez C Mora N Aleman OR Uribe-Querol E Carrero JC. Entamoeba Histolytica Induce Signaling *via* Raf/MEK/ERK for Neutrophil Extracellular Trap (NET) Formation. Front Cell Infect Microbiol (2018) 8:226. 10.3389/fcimb.2018.00226 30023352 PMC6039748

[46] Yin K Yang Z Gong Y Wang D Lin H. The Antagonistic Effect of Se on the Pb-Weakening Formation of Neutrophil Extracellular Traps in Chicken Neutrophils. Ecotoxicol Environ Saf (2019) 173:225–34. 10.1016/j.ecoenv.2019.02.033 30772712

[47] Hu Q Shi H Zeng T Liu H Su Y Cheng X. Increased Neutrophil Extracellular Traps Activate NLRP3 and Inflammatory Macrophages in Adult-Onset Still’s Disease. Arthritis Res Ther (2019) 21:9. 10.1186/s13075-018-1800-z 30616678 PMC6323819

[48] Liu D Yang P Gao M Yu T Shi Y Zhang M. NLRP3 Activation Induced by Neutrophil Extracellular Traps Sustains Inflammatory Response in the Diabetic Wound. Clin Sci (Lond) (2019) 133:565–82. 10.1042/CS20180600 30626731

[49] Skendros P Mitroulis I Ritis K. Autophagy in Neutrophils: From Granulopoiesis to Neutrophil Extracellular Traps. Front Cell Dev Biol (2018) 6:109. 10.3389/fcell.2018.00109 30234114 PMC6131573

[50] An Z Li J Yu J Wang X Gao H Zhang W. Neutrophil Extracellular Traps Induced by IL-8 Aggravate Atherosclerosis *via* Activation NF-kappaB Signaling in Macrophages. Cell Cycle (2019) 18:2928–38. 10.1080/15384101.2019.1662678 PMC6791689 31496351

[51] Gonzalez-Aparicio M Alfaro C. Influence of Interleukin-8 and Neutrophil Extracellular Trap (NET) Formation in the Tumor Microenvironment: Is There a Pathogenic Role? J Immunol Res (2019) 2019:6252138. 10.1155/2019/6252138 31093511 PMC6481028

[52] Wang S Zheng S Zhang Q Yang Z Yin K Xu S. Atrazine Hinders PMA-Induced Neutrophil Extracellular Traps in Carp *via* the Promotion of Apoptosis and Inhibition of ROS Burst, Autophagy and Glycolysis. Environ Pollut (2018) 243:282–91. 10.1016/j.envpol.2018.08.070 30193222

[53] Galkina SI Fedorova NV Golenkina EA Stadnichuk VI Sud’ina GF. Cytonemes *Versus* Neutrophil Extracellular Traps in the Fight of Neutrophils With Microbes. Int J Mol Sci (2020) 21:586. 10.3390/ijms21020586 PMC7014225 31963289

[54] Cox LE Walstein K Vollger L Reuner F Bick A Dotsch A. Neutrophil Extracellular Trap Formation and Nuclease Activity in Septic Patients. BMC Anesthesiol (2020) 20:15. 10.1186/s12871-019-0911-7 31931719 PMC6958610

[55] Colon DF Wanderley CW Franchin M Silva CM Hiroki CH Castanheira FVS. Neutrophil Extracellular Traps (NETs) Exacerbate Severity of Infant Sepsis. Crit Care (2019) 23:113. 10.1186/s13054-019-2407-8 30961634 PMC6454713

[56] Arroyo R Khan MA Echaide M Perez-Gil J Palaniyar N. SP-D Attenuates LPS-Induced Formation of Human Neutrophil Extracellular Traps (NETs), Protecting Pulmonary Surfactant Inactivation by NETs. Commun Biol (2019) 2:470. 10.1038/s42003-019-0662-5 31872075 PMC6915734

[57] Clark SR Ma AC Tavener SA McDonald B Goodarzi Z Kelly MM. Platelet TLR4 Activates Neutrophil Extracellular Traps to Ensnare Bacteria in Septic Blood. Nat Med (2007) 13:463–9. 10.1038/nm1565 17384648

[58] Rada B. Neutrophil Extracellular Traps. Methods Mol Biol (2019) 1982:517–28. 10.1007/978-1-4939-9424-3_31 PMC6874304 31172493

[59] Urban CF Nett JE. Neutrophil Extracellular Traps in Fungal Infection. Semin Cell Dev Biol (2019) 89:47–57. 10.1016/j.semcdb.2018.03.020 29601861 PMC6170733

[60] Dinallo V Marafini I Di Fusco D Laudisi F Franze E Di Grazia A. Neutrophil Extracellular Traps Sustain Inflammatory Signals in Ulcerative Colitis. J Crohns Colitis (2019) 13:772–84. 10.1093/ecco-jcc/jjy215 30715224

[61] Folco EJ Mawson TL Vromman A Bernardes-Souza B Franck G Persson O. Neutrophil Extracellular Traps Induce Endothelial Cell Activation and Tissue Factor Production Through Interleukin-1alpha and Cathepsin G. Arterioscler Thromb Vasc Biol (2018) 38:1901–12. 10.1161/ATVBAHA.118.311150 PMC6202190 29976772

[62] Laridan E Martinod K De Meyer SF. Neutrophil Extracellular Traps in Arterial and Venous Thrombosis. Semin Thromb Hemost (2019) 45:86–93. 10.1055/s-0038-1677040 30634198

[63] Gao H Wang X Lin C An Z Yu J Cao H. Exosomal MALAT1 Derived From Ox-LDL-Treated Endothelial Cells Induce Neutrophil Extracellular Traps to Aggravate Atherosclerosis. Biol Chem (2020) 401:367–76. 10.1515/hsz-2019-0219 31318684

[64] Wang YP Guo Y Wen PS Zhao ZZ Xie J Yang K. Three Ingredients of Safflower Alleviate Acute Lung Injury and Inhibit NET Release Induced by Lipopolysaccharide. Mediators Inflamm (2020) 2020:2720369. 10.1155/2020/2720369 32189992 PMC7066412

[65] Wei Z Wang J Wang Y Wang C Liu X Han Z. Effects of Neutrophil Extracellular Traps on Bovine Mammary Epithelial Cells *In Vitro* . Front Immunol (2019) 10:1003. 10.3389/fimmu.2019.01003 31156617 PMC6533846

[66] Li T Wang C Liu Y Li B Zhang W Wang L. Neutrophil Extracellular Traps Induce Intestinal Damage and Thrombotic Tendency in Inflammatory Bowel Disease. J Crohns Colitis (2020) 14:240–53. 10.1093/ecco-jcc/jjz132 31325355

[67] Kubiritova Z Radvanszky J Gardlik R. Cell-Free Nucleic Acids and Their Emerging Role in the Pathogenesis and Clinical Management of Inflammatory Bowel Disease. Int J Mol Sci (2019) 20:3662. 10.3390/ijms20153662 PMC6696129 31357438

[68] Gottlieb Y Elhasid R Berger-Achituv S Brazowski E Yerushalmy-Feler A Cohen S. Neutrophil Extracellular Traps in Pediatric Inflammatory Bowel Disease. Pathol Int (2018) 68:517–23. 10.1111/pin.12715 30133056

[69] Manda-Handzlik A Demkow U. The Brain Entangled: The Contribution of Neutrophil Extracellular Traps to the Diseases of the Central Nervous System. Cells (2019) 8:1477. 10.3390/cells8121477 PMC6953104 31766346

[70] Paryzhak S Dumych T Mahorivska I Boichuk M Bila G Peshkova S. Neutrophil-Released Enzymes can Influence Composition of Circulating Immune Complexes in Multiple Sclerosis. Autoimmunity (2018) 51:297–303. 10.1080/08916934.2018.1514390 30369266

[71] Josefs T Barrett TJ Brown EJ Quezada A Wu X Voisin M. Neutrophil Extracellular Traps Promote Macrophage Inflammation and Impair Atherosclerosis Resolution in Diabetic Mice. JCI Insight (2020) 5:e134796. 10.1172/jci.insight.134796 PMC7205252 32191637

[72] Doring Y Libby P Soehnlein O. Neutrophil Extracellular Traps Participate in Cardiovascular Diseases: Recent Experimental and Clinical Insights. Circ Res (2020) 126:1228–41. 10.1161/CIRCRESAHA.120.315931 PMC7185047 32324499

[73] Ducroux C Di Meglio L Loyau S Delbosc S Boisseau W Deschildre C. Thrombus Neutrophil Extracellular Traps Content Impair tPA-Induced Thrombolysis in Acute Ischemic Stroke. Stroke (2018) 49:754–57. 10.1161/STROKEAHA.117.019896 29438080

[74] Granger V Peyneau M Chollet-Martin S de Chaisemartin L. Neutrophil Extracellular Traps in Autoimmunity and Allergy: Immune Complexes at Work. Front Immunol (2019) 10:2824. 10.3389/fimmu.2019.02824 31849989 PMC6901596

[75] Demers M Krause DS Schatzberg D Martinod K Voorhees JR Fuchs TA. Cancers Predispose Neutrophils to Release Extracellular DNA Traps That Contribute to Cancer-Associated Thrombosis. Proc Natl Acad Sci U S A (2012) 109:13076–81. 10.1073/pnas.1200419109 PMC3420209 22826226

[76] Houghton AM Rzymkiewicz DM Ji H Gregory AD Egea EE Metz HE. Neutrophil Elastase-Mediated Degradation of IRS-1 Accelerates Lung Tumor Growth. Nat Med (2010) 16:219–23. 10.1038/nm.2084 PMC2821801 20081861

[77] Gregory AD Hale P Perlmutter DH Houghton AM. Clathrin Pit-Mediated Endocytosis of Neutrophil Elastase and Cathepsin G by Cancer Cells. J Biol Chem (2012) 287:35341–50. 10.1074/jbc.M112.385617 PMC3471748 22915586

[78] Acuff HB Carter KJ Fingleton B Gorden DL Matrisian LM. Matrix Metalloproteinase-9 From Bone Marrow-Derived Cells Contributes to Survival But Not Growth of Tumor Cells in the Lung Microenvironment. Cancer Res (2006) 66:259–66. 10.1158/0008-5472.CAN-05-2502 PMC1360653 16397239

[79] Teijeira A Garasa S Gato M Alfaro C Migueliz I Cirella A. CXCR1 and CXCR2 Chemokine Receptor Agonists Produced by Tumors Induce Neutrophil Extracellular Traps That Interfere With Immune Cytotoxicity. Immunity (2020) 52:856–71.e8. 10.1016/j.immuni.2020.03.001 32289253

[80] Ireland AS Oliver TG. Neutrophils Create an ImpeNETrable Shield Between Tumor and Cytotoxic Immune Cells. Immunity (2020) 52:729–31. 10.1016/j.immuni.2020.04.009 PMC7851833 32433945

[81] Yazdani HO Roy E Comerci AJ van der Windt DJ Zhang H Huang H. Neutrophil Extracellular Traps Drive Mitochondrial Homeostasis in Tumors to Augment Growth. Cancer Res (2019) 79:5626–39. 10.1158/0008-5472.CAN-19-0800 PMC6825588 31519688

[82] Stoiber D Assinger A. Platelet-Leukocyte Interplay in Cancer Development and Progression. Cells (2020) 9:855. 10.3390/cells9040855 PMC7226828 32244723

[83] Mansour A Bachelot-Loza C Nesseler N Gaussem P Gouin-Thibault I. P2Y12 Inhibition Beyond Thrombosis: Effects on Inflammation. Int J Mol Sci (2020) 21:1391. 10.3390/ijms21041391 PMC7073040 32092903

[84] Schlesinger M. Role of Platelets and Platelet Receptors in Cancer Metastasis. J Hematol Oncol (2018) 11:125. 10.1186/s13045-018-0669-2 30305116 PMC6180572

[85] Almeida VH Rondon AMR Gomes T Monteiro RQ. Novel Aspects of Extracellular Vesicles as Mediators of Cancer-Associated Thrombosis. Cells (2019) 8:716. 10.3390/cells8070716 PMC6679024 31337034

[86] Rayes RF Mouhanna JG Nicolau I Bourdeau F Giannias B Rousseau S. Primary Tumors Induce Neutrophil Extracellular Traps With Targetable Metastasis Promoting Effects. JCI Insight (2019) 5:e128008. 10.1172/jci.insight.128008 PMC6777835 31343990

[87] Takesue S Ohuchida K Shinkawa T Otsubo Y Matsumoto S Sagara A. Neutrophil Extracellular Traps Promote Liver Micrometastasis in Pancreatic Ductal Adenocarcinoma *via* the Activation of Cancerassociated Fibroblasts. Int J Oncol (2020) 56:596–605. 10.3892/ijo.2019.4951 31894273

[88] Gregoire M Guilloton F Pangault C Mourcin F Sok P Latour M. Neutrophils Trigger a NF-kappaB Dependent Polarization of Tumor-Supportive Stromal Cells in Germinal Center B-Cell Lymphomas. Oncotarget (2015) 6:16471–87. 10.18632/oncotarget.4106 PMC4599283 26158216

[89] Presta M Chiodelli P Giacomini A Rusnati M Ronca R. Fibroblast Growth Factors (FGFs) in Cancer: FGF Traps as a New Therapeutic Approach. Pharmacol Ther (2017) 179:171–87. 10.1016/j.pharmthera.2017.05.013 28564583

[90] Liu Y Liu L. The Pro-Tumor Effect and the Anti-Tumor Effect of Neutrophils Extracellular Traps. Biosci Trends (2020) 13:469–75. 10.5582/bst.2019.01326 31866615

[91] Triner D Devenport SN Ramakrishnan SK Ma X Frieler RA Greenson JK. Neutrophils Restrict Tumor-Associated Microbiota to Reduce Growth and Invasion of Colon Tumors in Mice. Gastroenterology (2019) 156:1467–82. 10.1053/j.gastro.2018.12.003 PMC6441634 30550822

[92] Zhou J Yang Y Gan T Li Y Hu F Hao N. Lung Cancer Cells Release High Mobility Group Box 1 and Promote the Formation of Neutrophil Extracellular Traps. Oncol Lett (2019) 18:181–8. 10.3892/ol.2019.10290 PMC6540031 31289487

[93] Yang LY Luo Q Lu L Zhu WW Sun HT Wei R. Increased Neutrophil Extracellular Traps Promote Metastasis Potential of Hepatocellular Carcinoma *via* Provoking Tumorous Inflammatory Response. J Hematol Oncol (2020) 13:3. 10.1186/s13045-019-0836-0 31907001 PMC6945602

[94] Scandolara TB Panis C. Neutrophil Traps, Anti-Myeloperoxidase Antibodies and Cancer: Are They Linked? Immunol Lett (2020) 221:33–8. 10.1016/j.imlet.2020.02.011 32092357

[95] Evavold CL Kagan JC. Inflammasomes: Threat-Assessment Organelles of the Innate Immune System. Immunity (2019) 51:609–24. 10.1016/j.immuni.2019.08.005 PMC6801093 31473100

[96] Shao BZ Wang SL Pan P Yao J Wu K Li ZS. Targeting NLRP3 Inflammasome in Inflammatory Bowel Disease: Putting Out the Fire of Inflammation. Inflammation (2019) 42:1147–59. 10.1007/s10753-019-01008-y 30937839

[97] Schroder K Tschopp J. The Inflammasomes. Cell (2010) 140:821–32. 10.1016/j.cell.2010.01.040 20303873

[98] He Y Zeng MY Yang D Motro B Nunez G. NEK7 is an Essential Mediator of NLRP3 Activation Downstream of Potassium Efflux. Nature (2016) 530:354–7. 10.1038/nature16959 PMC4810788 26814970

[99] Shao BZ Xu ZQ Han BZ Su DF Liu C. NLRP3 Inflammasome and its Inhibitors: A Review. Front Pharmacol (2015) 6:262. 10.3389/fphar.2015.00262 26594174 PMC4633676

[100] Shao BZ Cao Q Liu C. Targeting NLRP3 Inflammasome in the Treatment of CNS Diseases. Front Mol Neurosci (2018) 11:320. 10.3389/fnmol.2018.00320 30233319 PMC6131647

[101] Shao BZ Wei W Ke P Xu ZQ Zhou JX Liu C. Activating Cannabinoid Receptor 2 Alleviates Pathogenesis of Experimental Autoimmune Encephalomyelitis *via* Activation of Autophagy and Inhibiting NLRP3 Inflammasome. CNS Neurosci Ther (2014) 20:1021–8. 10.1111/cns.12349 PMC6492996 25417929

[102] Ke P Shao BZ Xu ZQ Chen XW Wei W Liu C. Activating Alpha7 Nicotinic Acetylcholine Receptor Inhibits NLRP3 Inflammasome Through Regulation of Beta-Arrestin-1. CNS Neurosci Ther (2017) 23:875–84. 10.1111/cns.12758 PMC6492746 28941191

[103] Rathinam VA Fitzgerald KA. Inflammasome Complexes: Emerging Mechanisms and Effector Functions. Cell (2016) 165:792–800. 10.1016/j.cell.2016.03.046 27153493 PMC5503689

[104] Karki R Man SM Kanneganti TD. Inflammasomes and Cancer. Cancer Immunol Res (2017) 5:94–9. 10.1158/2326-6066.CIR-16-0269 PMC5593081 28093447

[105] Cao X Xu J. Insights Into Inflammasome and its Research Advances in Cancer. Tumori (2019) 105:456–64. 10.1177/0300891619868007 31407634

[106] Van Gorp H Lamkanfi M. The Emerging Roles of Inflammasome-Dependent Cytokines in Cancer Development. EMBO Rep (2019) 20:e47575. 10.15252/embr.201847575 31101676 PMC6549028

[107] Guo B Fu S Zhang J Liu B Li Z. Targeting Inflammasome/IL-1 Pathways for Cancer Immunotherapy. Sci Rep (2016) 6:36107. 10.1038/srep36107 27786298 PMC5082376

[108] Huang CF Chen L Li YC Wu L Yu GT Zhang WF. NLRP3 Inflammasome Activation Promotes Inflammation-Induced Carcinogenesis in Head and Neck Squamous Cell Carcinoma. J Exp Clin Cancer Res (2017) 36:116. 10.1186/s13046-017-0589-y 28865486 PMC5581464

[109] Wang H Luo Q Feng X Zhang R Li J Chen F. NLRP3 Promotes Tumor Growth and Metastasis in Human Oral Squamous Cell Carcinoma. BMC Cancer (2018) 18:500. 10.1186/s12885-018-4403-9 29716544 PMC5930757

[110] Ershaid N Sharon Y Doron H Raz Y Shani O Cohen N. NLRP3 Inflammasome in Fibroblasts Links Tissue Damage With Inflammation in Breast Cancer Progression and Metastasis. Nat Commun (2019) 10:4375. 10.1038/s41467-019-12370-8 31558756 PMC6763472

[111] Lachowicz-Scroggins ME Dunican EM Charbit AR Raymond W Looney MR Peters MC. Extracellular DNA, Neutrophil Extracellular Traps, and Inflammasome Activation in Severe Asthma. Am J Respir Crit Care Med (2019) 199:1076–85. 10.1164/rccm.201810-1869OC PMC6515873 30888839

[112] Warnatsch A Ioannou M Wang Q Papayannopoulos V. Inflammation. Neutrophil Extracellular Traps License Macrophages for Cytokine Production in Atherosclerosis. Science (2015) 349:316–20. 10.1126/science.aaa8064 PMC4854322 26185250

[113] Elssner A Duncan M Gavrilin M Wewers MD. A Novel P2X7 Receptor Activator, the Human Cathelicidin-Derived Peptide LL37, Induces IL-1 Beta Processing and Release. J Immunol (2004) 172:4987–94. 10.4049/jimmunol.172.8.4987 15067080

[114] Meyer-Hoffert U Wiedow O. Neutrophil Serine Proteases: Mediators of Innate Immune Responses. Curr Opin Hematol (2011) 18:19–24. 10.1097/MOH.0b013e32834115d1 21042214

[115] Jorgensen I Lopez JP Laufer SA Miao EA. IL-1beta, IL-18, and Eicosanoids Promote Neutrophil Recruitment to Pore-Induced Intracellular Traps Following Pyroptosis. Eur J Immunol (2016) 46:2761–66. 10.1002/eji.201646647 PMC5138142 27682622

[116] Yim WW Mizushima N. Lysosome Biology in Autophagy. Cell Discov (2020) 6:6. 10.1038/s41421-020-0141-7 32047650 PMC7010707

[117] Chang NC. Autophagy and Stem Cells: Self-Eating for Self-Renewal. Front Cell Dev Biol (2020) 8:138. 10.3389/fcell.2020.00138 32195258 PMC7065261

[118] Allen EA Baehrecke EH. Autophagy in Animal Development. Cell Death Differ (2020) 27:903–18. 10.1038/s41418-020-0497-0 PMC7206001 31988494

[119] Wang P Shao BZ Deng Z Chen S Yue Z Miao CY. Autophagy in Ischemic Stroke. Prog Neurobiol (2018) 163-164:98–117. 10.1016/j.pneurobio.2018.01.001 29331396

[120] Shao BZ Han BZ Zeng YX Su DF Liu C. The Roles of Macrophage Autophagy in Atherosclerosis. Acta Pharmacol Sin (2016) 37:150–6. 10.1038/aps.2015.87 PMC4753375 26750103

[121] Wang SL Shao BZ Zhao SB Fang J Gu L Miao CY. Impact of Paneth Cell Autophagy on Inflammatory Bowel Disease. Front Immunol (2018) 9:693. 10.3389/fimmu.2018.00693 29675025 PMC5895641

[122] Van Noorden R Ledford H. Medicine Nobel for Research on How Cells ‘Eat Themselves’. Nature (2016) 538:18–9. 10.1038/nature.2016.20721 27708326

[123] Klionsky DJ Abdelmohsen K Abe A Abedin MJ Abeliovich H Acevedo Arozena A. Guidelines for the Use and Interpretation of Assays for Monitoring Autophagy (3rd Edition). Autophagy (2016) 12:1–222. 10.1080/15548627.2015.1100356 26799652 PMC4835977

[124] Wang SL Shao BZ Zhao SB Chang X Wang P Miao CY. Intestinal Autophagy Links Psychosocial Stress With Gut Microbiota to Promote Inflammatory Bowel Disease. Cell Death Dis (2019) 10:391. 10.1038/s41419-019-1634-x 31564717 PMC6766473

[125] Shao BZ Wang SL Fang J Li ZS Bai Y Wu K. Alpha7 Nicotinic Acetylcholine Receptor Alleviates Inflammatory Bowel Disease Through Induction of AMPK-mTOR-P70s6k-Mediated Autophagy. Inflammation (2019) 42:1666–79. 10.1007/s10753-019-01027-9 31236857

[126] Shao BZ Ke P Xu ZQ Wei W Cheng MH Han BZ. Autophagy Plays an Important Role in Anti-Inflammatory Mechanisms Stimulated by Alpha7 Nicotinic Acetylcholine Receptor. Front Immunol (2017) 8:553. 10.3389/fimmu.2017.00553 28559895 PMC5432615

[127] Glick D Barth S Macleod KF. Autophagy: Cellular and Molecular Mechanisms. J Pathol (2010) 221:3–12. 10.1002/path.2697 20225336 PMC2990190

[128] Parzych KR Klionsky DJ. An Overview of Autophagy: Morphology, Mechanism, and Regulation. Antioxid Redox Signal (2014) 20:460–73. 10.1089/ars.2013.5371 PMC3894687 23725295

[129] Ravanan P Srikumar IF Talwar P. Autophagy: The Spotlight for Cellular Stress Responses. Life Sci (2017) 188:53–67. 10.1016/j.lfs.2017.08.029 28866100

[130] Onorati AV Dyczynski M Ojha R Amaravadi RK. Targeting Autophagy in Cancer. Cancer (2018) 124:3307–18. 10.1002/cncr.31335 PMC6108917 29671878

[131] Yun CW Lee SH. The Roles of Autophagy in Cancer. Int J Mol Sci (2018) 19:3466. 10.3390/ijms19113466 PMC6274804 30400561

[132] Fu Y Gu Q Luo L Xu J Luo Y Xia F. New Anti-Cancer Strategy to Suppress Colorectal Cancer Growth Through Inhibition of ATG4B and Lysosome Function. Cancers (Basel) (2020) 12:1523. 10.3390/cancers12061523 PMC7352571 32532053

[133] Bian Y Zeng H Tao H Huang L Du Z Wang J. A Pectin-Like Polysaccharide From Polygala Tenuifolia Inhibits Pancreatic Cancer Cell Growth *In Vitro* and *In Vivo* by Inducing Apoptosis and Suppressing Autophagy. Int J Biol Macromol (2020) 162:107–15. 10.1016/j.ijbiomac.2020.06.054 32531363

[134] Jiang H Chen H Jin C Mo J Wang H. Nobiletin Flavone Inhibits the Growth and Metastasis of Human Pancreatic Cancer Cells *via* Induction of Autophagy, G0/G1 Cell Cycle Arrest and Inhibition of NF-kB Signalling Pathway. J BUON (2020) 25:1070–75. 32521908

[135] Ma R Li T Cao M Si Y Wu X Zhao L. Extracellular DNA Traps Released by Acute Promyelocytic Leukemia Cells Through Autophagy. Cell Death Dis (2016) 7:e2283. 10.1038/cddis.2016.186 27362801 PMC5108337

[136] Boone BA Orlichenko L Schapiro NE Loughran P Gianfrate GC Ellis JT. The Receptor for Advanced Glycation End Products (RAGE) Enhances Autophagy and Neutrophil Extracellular Traps in Pancreatic Cancer. Cancer Gene Ther (2015) 22:326–34. 10.1038/cgt.2015.21 PMC4470814 25908451

[137] Boone BA Murthy P Miller-Ocuin J Doerfler WR Ellis JT Liang X. Chloroquine Reduces Hypercoagulability in Pancreatic Cancer Through Inhibition of Neutrophil Extracellular Traps. BMC Cancer (2018) 18:678. 10.1186/s12885-018-4584-2 29929491 PMC6013899

[138] Lin CF Chien SY Chen CL Hsieh CY Tseng PC Wang YC. IFN-Gamma Induces Mimic Extracellular Trap Cell Death in Lung Epithelial Cells Through Autophagy-Regulated DNA Damage. J Interferon Cytokine Res (2016) 36:100–12. 10.1089/jir.2015.0011 26540174

[139] Shi L Yao H Liu Z Xu M Tsung A Wang Y. Endogenous PAD4 in Breast Cancer Cells Mediates Cancer Extracellular Chromatin Network Formation and Promotes Lung Metastasis. Mol Cancer Res (2020) 18:735–47. 10.1158/1541-7786.MCR-19-0018 PMC7668292 32193354

[140] Martins-Cardoso K Almeida VH Bagri KM Rossi MID Mermelstein CS Konig S. Neutrophil Extracellular Traps (NETs) Promote Pro-Metastatic Phenotype in Human Breast Cancer Cells Through Epithelial-Mesenchymal Transition. Cancers (Basel) (2020) 12:735–47. 10.3390/cancers12061542 PMC7352979 32545405

[141] Yang L Liu Q Zhang X Liu X Zhou B Chen J. DNA of Neutrophil Extracellular Traps Promotes Cancer Metastasis *via* CCDC25. Nature (2020) 12:1542. 10.1038/s41586-020-2394-6 32528174

[142] Gomes T Varady CBS Lourenco AL Mizurini DM Rondon AMR Leal AC. IL-1beta Blockade Attenuates Thrombosis in a Neutrophil Extracellular Trap-Dependent Breast Cancer Model. Front Immunol (2019) 10:2088. 10.3389/fimmu.2019.02088 31552036 PMC6737452

[143] Li Y Yang Y Gan T Zhou J Hu F Hao N. Extracellular RNAs From Lung Cancer Cells Activate Epithelial Cells and Induce Neutrophil Extracellular Traps. Int J Oncol (2019) 55:69–80. 10.3892/ijo.2019.4808 31115506 PMC6561626

[144] Li Z Lin Y Zhang S Zhou L Yan G Wang Y. Emodin Regulates Neutrophil Phenotypes to Prevent Hypercoagulation and Lung Carcinogenesis. J Transl Med (2019) 17:90. 10.1186/s12967-019-1838-y 30885207 PMC6423780

[145] Zhang Y Wang C Yu M Zhao X Du J Li Y. Neutrophil Extracellular Traps Induced by Activated Platelets Contribute to Procoagulant Activity in Patients With Colorectal Cancer. Thromb Res (2019) 180:87–97. 10.1016/j.thromres.2019.06.005 31271975

[146] Rayes RF Vourtzoumis P Bou Rjeily M Seth R Bourdeau F Giannias B. Neutrophil Extracellular Trap-Associated CEACAM1 as a Putative Therapeutic Target to Prevent Metastatic Progression of Colon Carcinoma. J Immunol (2020) 204:2285–94. 10.4049/jimmunol.1900240 PMC7534954 32169849

[147] Shang A Gu C Zhou C Yang Y Chen C Zeng B. Exosomal KRAS Mutation Promotes the Formation of Tumor-Associated Neutrophil Extracellular Traps and Causes Deterioration of Colorectal Cancer by Inducing IL-8 Expression. Cell Commun Signal (2020) 18:52. 10.1186/s12964-020-0517-1 32228650 PMC7106821

[148] Yang L Liu L Zhang R Hong J Wang Y Wang J. IL-8 Mediates a Positive Loop Connecting Increased Neutrophil Extracellular Traps (NETs) and Colorectal Cancer Liver Metastasis. J Cancer (2020) 11:4384–96. 10.7150/jca.44215 PMC7255375 32489457

[149] Yu M Li T Li B Liu Y Wang L Zhang J. Phosphatidylserine-Exposing Blood Cells, Microparticles and Neutrophil Extracellular Traps Increase Procoagulant Activity in Patients With Pancreatic Cancer. Thromb Res (2020) 188:5–16. 10.1016/j.thromres.2020.01.025 32032826

[150] Jin W Yin H Li H Yu XJ Xu HX Liu L. Neutrophil Extracellular DNA Traps Promote Pancreatic Cancer Cells Migration and Invasion by Activating EGFR/ERK Pathway. J Cell Mol Med (2021) 25:5443–56. 10.1111/jcmm.16555 PMC8184670 33955688

[151] Li M Lin C Deng H Strnad J Bernabei L Vogl DT. A Novel Peptidylarginine Deiminase 4 (PAD4) Inhibitor BMS-P5 Blocks Formation of Neutrophil Extracellular Traps and Delays Progression of Multiple Myeloma. Mol Cancer Ther (2020) 180:87–97. 10.1158/1535-7163.MCT-19-1020 PMC7335350 32371579

[152] Craver BM Ramanathan G Hoang S Chang X Mendez Luque LF Brooks S. N-Acetylcysteine Inhibits Thrombosis in a Murine Model of Myeloproliferative Neoplasm. Blood Adv (2020) 4:312–21. 10.1182/bloodadvances.2019000967 PMC6988398 31978215

[153] Wolach O Sellar RS Martinod K Cherpokova D McConkey M Chappell RJ. Increased Neutrophil Extracellular Trap Formation Promotes Thrombosis in Myeloproliferative Neoplasms. Sci Transl Med (2018) 10:52. 10.1126/scitranslmed.aan8292 PMC6442466 29643232

[154] Risnik D Elias EE Keitelman I Colado A Podaza E Cordini G. The Effect of Ibrutinib on Neutrophil and Gammadelta T Cell Functions. Leuk Lymphoma (2020) 11(15):4384–96. 10.1080/10428194.2020.1753043 32306816

[155] Thalin C Demers M Blomgren B Wong SL von Arbin M von Heijne A. NETosis Promotes Cancer-Associated Arterial Microthrombosis Presenting as Ischemic Stroke With Troponin Elevation. Thromb Res (2016) 139:56–64. 10.1016/j.thromres.2016.01.009 26916297 PMC4769435

[156] Zha C Meng X Li L Mi S Qian D Li Z. Neutrophil Extracellular Traps Mediate the Crosstalk Between Glioma Progression and the Tumor Microenvironment *via* the HMGB1/RAGE/IL-8 Axis. Cancer Biol Med (2020) 17:154–68. 10.20892/j.issn.2095-3941.2019.0353 PMC7142852 32296583

[157] Blenman KRM Wang J Cowper S Bosenberg M. Pathology of Spontaneous and Immunotherapy-Induced Tumor Regression in a Murine Model of Melanoma. Pigment Cell Melanoma Res (2019) 32:448–57. 10.1111/pcmr.12769 PMC6500596 30702217

[158] Schedel F Mayer-Hain S Pappelbaum KI Metze D Stock M Goerge T. Evidence and Impact of Neutrophil Extracellular Traps in Malignant Melanoma. Pigment Cell Melanoma Res (2020) 33:63–73. 10.1111/pcmr.12818 31402559

[159] Andzinski L Kasnitz N Stahnke S Wu CF Gereke M von Kockritz-Blickwede M. Type I IFNs Induce Anti-Tumor Polarization of Tumor Associated Neutrophils in Mice and Human. Int J Cancer (2016) 138:1982–93. 10.1002/ijc.29945 26619320

[160] Harbeck N Gnant M. Breast Cancer. Lancet (2017) 389:1134–50. 10.1016/S0140-6736(16)31891-8 27865536

[161] Hirsch FR Scagliotti GV Mulshine JL Kwon R Curran WJ Jr Wu YL. Lung Cancer: Current Therapies and New Targeted Treatments. Lancet (2017) 389:299–311. 10.1016/S0140-6736(16)30958-8 27574741

[162] Arpinati L Shaul ME Kaisar-Iluz N Mali S Mahroum S Fridlender ZG. NETosis in Cancer: A Critical Analysis of the Impact of Cancer on Neutrophil Extracellular Trap (NET) Release in Lung Cancer Patients *vs.* Mice. Cancer Immunol Immunother (2020) 69:199–213. 10.1007/s00262-019-02474-x 31982939 PMC11027875

[163] Dekker E Tanis PJ Vleugels JLA Kasi PM Wallace MB. Colorectal Cancer. Lancet (2019) 394:1467–80. 10.1016/S0140-6736(19)32319-0 31631858

[164] Brenner H Kloor M Pox CP. Colorectal Cancer. Lancet (2014) 383:1490–502. 10.1016/S0140-6736(13)61649-9 24225001

[165] Richardson JJR Hendrickse C Gao-Smith F Thickett DR. Neutrophil Extracellular Trap Production in Patients With Colorectal Cancer *In Vitro* . Int J Inflamm (2017) 2017:4915062. 10.1155/2017/4915062 PMC5554570 28828191

[166] Kamisawa T Wood LD Itoi T Takaori K. Pancreatic Cancer. Lancet (2016) 388:73–85. 10.1016/S0140-6736(16)00141-0 26830752

[167] Jin W Xu HX Zhang SR Li H Wang WQ Gao HL. Tumor-Infiltrating NETs Predict Postsurgical Survival in Patients With Pancreatic Ductal Adenocarcinoma. Ann Surg Oncol (2019) 26:635–43. 10.1245/s10434-018-6941-4 30374923

[168] Campello E Ilich A Simioni P Key NS. The Relationship Between Pancreatic Cancer and Hypercoagulability: A Comprehensive Review on Epidemiological and Biological Issues. Br J Cancer (2019) 121:359–71. 10.1038/s41416-019-0510-x PMC6738049 31327867

[169] Hisada Y Grover SP Maqsood A Houston R Ay C Noubouossie DF. Neutrophils and Neutrophil Extracellular Traps Enhance Venous Thrombosis in Mice Bearing Human Pancreatic Tumors. Haematologica (2020) 105:218–25. 10.3324/haematol.2019.217083 PMC6939515 31048354

[170] Kennedy A. Make Blood Cancer Visible. Lancet Oncol (2017) 18:1577. 10.1016/S1470-2045(17)30849-5 29208430

[171] Fagerhol MK Johnson E Tangen JM Hollan I Mirlashari MR Nissen-Meyer LSH. NETs Analysed by Novel Calprotectin-Based Assays in Blood Donors and Patients With Multiple Myeloma or Rheumatoid Arthritis: A Pilot Study. Scand J Immunol (2020) 91:e12870. 10.1111/sji.12870 32034957

[172] Balachandran AA Larcher LM Chen S Veedu RN. Therapeutically Significant MicroRNAs in Primary and Metastatic Brain Malignancies. Cancers (Basel) (2020) 12:645–59. 10.3390/cancers12092534 PMC7564168 32906592

[173] Louis DN Perry A Reifenberger G von Deimling A Figarella-Branger D Cavenee WK. The 2016 World Health Organization Classification of Tumors of the Central Nervous System: A Summary. Acta Neuropathol (2016) 131:803–20. 10.1007/s00401-016-1545-1 27157931

[174] Pabinger I Posch F. Flamethrowers: Blood Cells and Cancer Thrombosis Risk. Hematol Am Soc Hematol Educ Program (2014) 2014:410–7. 10.1182/asheducation-2014.1.410 25696887

[175] Linares MA Zakaria A Nizran P. Skin Cancer. Prim Care (2015) 42:645–59. 10.1016/j.pop.2015.07.006 26612377

[176] Marone G Schroeder JT Mattei F Loffredo S Gambardella AR Poto R. Is There a Role for Basophils in Cancer? Front Immunol (2020) 11:2103. 10.3389/fimmu.2020.02103 33013885 PMC7505934

[177] Mussbacher M Salzmann M Brostjan C Hoesel B Schoergenhofer C Datler H. Cell Type-Specific Roles of NF-kappaB Linking Inflammation and Thrombosis. Front Immunol (2019) 10:85. 10.3389/fimmu.2019.00085 30778349 PMC6369217

